# Generation of a panel of antibodies against proteins encoded on human chromosome 21

**DOI:** 10.1186/1477-5751-9-7

**Published:** 2010-08-20

**Authors:** Frances K Wiseman, Olivia Sheppard, Jacqueline M Linehan, Sebastian Brandner, Victor LJ Tybulewicz, Elizabeth MC Fisher

**Affiliations:** 1 Department of Neurodegenerative Disease, UCL Institute of Neurology, Queen Square, London WC1N 3BG, UK; 2 MRC National Institute for Medical Research, The Ridgeway, Mill Hill, London NW7 1AA, UK

## Abstract

**Background:**

Down syndrome (DS) is caused by trisomy of all or part of chromosome 21. To further understanding of DS we are working with a mouse model, the Tc1 mouse, which carries most of human chromosome 21 in addition to the normal mouse chromosome complement. This mouse is a model for human DS and recapitulates many of the features of the human syndrome such as specific heart defects, and cerebellar neuronal loss. The Tc1 mouse is mosaic for the human chromosome such that not all cells in the model carry it. Thus to help our investigations we aimed to develop a method to identify cells that carry human chromosome 21 in the Tc1 mouse. To this end, we have generated a panel of antibodies raised against proteins encoded by genes on human chromosome 21 that are known to be expressed in the adult brain of Tc1 mice

**Results:**

We attempted to generate human specific antibodies against proteins encoded by human chromosome 21. We selected proteins that are expressed in the adult brain of Tc1 mice and contain regions of moderate/low homology with the mouse ortholog. We produced antibodies to seven human chromosome 21 encoded proteins. Of these, we successfully generated three antibodies that preferentially recognise human compared with mouse SOD1 and RRP1 proteins on western blots. However, these antibodies did not specifically label cells which carry a freely segregating copy of Hsa21 in the brains of our Tc1 mouse model of DS.

**Conclusions:**

Although we have successfully isolated new antibodies to SOD1 and RRP1 for use on western blots, in our hands these antibodies have not been successfully used for immunohistochemistry studies. These antibodies are freely available to other researchers. Our data high-light the technical difficulty of producing species-specific antibodies for both western blotting and immunohistochemistry.

## Background

Down syndrome (DS) is the most common genetic cause of intellectual disability and is also associated with a number of other medical problems including heart defects, early onset Alzheimer's disease and leukaemia [1]. DS is caused by trisomy of human chromosome 21 and is a complex genetic disorder in which the phenotype arises from abnormal dosage of otherwise normal genes.

In order to investigate the relationship between phenotype and causative dosage sensitive genes in DS, we created the Tc1 mouse strain which carries a freely segregating copy of human chromosome 21 (Hsa21) in addition to a full complement of mouse chromosomes [2]. There are deletions in this Hsa21 [2] but at least 83% of the human genes are present in three copies (one human, two endogenous mouse homologs). Therefore, Tc1 mice are trisomic for the majority of genes on Hsa21 and several different investigations have shown they do indeed have phenotypes which are strikingly similar to those found in individuals with DS [2-5].

However, the Tc1 mouse is mosaic for Hsa21, owing to stochastic loss of the human chromosome in cells after fertilisation. Thus the mice have some cells that contain Hsa21 and some that are euploid, which have the normal mouse chromosome complement. The degree of mosaicism differs between tissues and is reported to vary between individual mice; in one survey carried out by genomic quantitative-PCR, on 8 animals, between 7 and 77% of cells in the brain of Tc1 mice carried the Hsa21 (mean 53%) [2]. When chromosome 21 content was assessed directly by fluorescence in situ hybridisation with a human specific probe on metaphase spreads of Tc1 brain cells, between 36 and 94% of the cells carried Hsa21 [2]. Between 2-4% of people with DS also have a mixture of euploid and trisomic cells [6,7]. A low proportion of trisomic cells in these individuals is associated with a reduced severity and incidence of DS associated phenotypes [8]. Additionally, people without DS have also been reported to be mosaic for Hsa21 trisomic cells, in particular individuals with Alzheimer's disease have been reported to have an elevated number of Hsa21 trisomic cells within their brains [9-11]. The phenotypic consequences of these observations have yet to be fully explored.

A study of Hsa21 mosaicism in the Tc1 mouse model may provide insight into these issues. In particular, variability in DS associated phenotypes observed in the Tc1 mouse model may result in part from variation in the number of Hsa21-containing cells in specific tissues and/or cell types. For example, only 73% of Tc1 mice show heart defects at E14.5, whereas the remaining 27% of their genetically identical, Hsa21 positive, littermates do not [2]. This may be due to variable penetrance of the effects of the dosage-sensitive Hsa21 genes, and/or it may be due to mosaicism in the hearts of these animals. In addition, if we could identify Hsa21 positive cells in vivo this may help us investigate the effects of Hsa21 trisomy at the cellular level. Therefore, in an effort to determine which cells in Tc1 mice carry Hsa21 and thus measure levels of mosaicism, we generated antibodies against proteins encoded by Hsa21 that do not cross react with mouse homologues. We focussed our study on proteins expressed in brain as this is our primary organ of interest.

We successfully generated antibodies that preferentially recognised human but not mouse forms of Hsa21-encoded proteins as shown by western blotting. However these antibodies were not compatible with immunohistochemical methods and therefore could not be used to identify individual cells that carry Hsa21. We note that these antibodies are available for other interested laboratories to use.

## Results

### Choice of candidate proteins

We aimed to generate novel human-specific antibodies raised against proteins encoded on Hsa21 to identify Hsa21 positive cells in our Tc1 mouse model of DS. Our principal goal was to produce a human-specific antibody that did not react with mouse proteins and that was highly expressed in the adult brain as this is our main organ of interest. We used published data and online resources (NCBI- Gene Expression Nervous System Atlas, Affymetrix Symatlas/BioGPS) to identify candidate genes that were reported to be expressed widely in the brain (Table 1). To avoid generating antibodies against hypothetical proteins we prioritised targets for which there was evidence of a functional protein. Regions of low homology between the human protein and the mouse homologue where then identified by performing Clustal W alignments. In the case of one gene, *ADARB1*, an exon unique to humans was identified.

**Table 1:** List of Hsa21 genes present in the Tc1 mouse that are expressed in adult brain.

Hsa21 encoded Protein	Evidence of expression in brain	Candidate regions and/or reasons for discontinuation
HSPA13/ STCH	Ubiquitous expressed [17]	No human specific region

NRIP1	Expressed in mouse brain [18] and Gensat images 24262 and 24263)	No human specific region

USP25	Basal expression in all human tissues but high expression only in fetal brain and adult testis [19,20]	N/A

NCAM2	Expression in adult human brain [21]	No human specific region

MRPL39	Expressed in adult mouse and human brain [18,21]	Possible region (aa 1-42 MEALAMGSRALRLWLVAPGGGIKWRFIATSSASQLSPTELTE) is putative mitochondrial targeting sequence [13]

JAM2	Expression in brain restricted to vascular endothelial cells [22,23]	N/A

GABPA	Expression in adult mouse brain [18]	No human specific region

ADAMTS5	Expression in adult mouse brain restricted to Schwann cells [24]	N/A

ADAMTS1	Expression in adult rat brain restricted to neuron subpopulation [25]	N/A

USP16	Expressed in human and mouse brain [18,21]	1 region used for antibody generation (see table 2).

CCT8/ CCTQ	Expressed in mouse brain [18,26,27]	No human specific region

BACH1	Expressed in adult mouse and human brain [21,28]	Possible region (aa 676-716 RPPAVLPPCARGNSEPGYARGQESQQMSTATSEQAGPAEQCR) contains a putative disulphide bond

GRIK1	Expressed in adult mouse and human brain [29,30]	No human specific region

TIAM1	Expression in adult mouse and human brain [18]	No human specific region

SOD1	Expressed in human and mouse brain [31,32]	1 region used for antibody generation (see table 2)

CBR1	Expressed in human adult and fetal brain [21,33]	No human specific region

CBR3	Expressed in human adult brain [34]	Possible region (aa 236-242 GKDSI) similarity with mouse Hy-3 and DNA isomerase 1

DOPEY2/C21orf5	Expression in cortex, cerebellum, and hippocampus in adult, widespread expression in embryonic and fetal brain [35-38]	1 region used for antibody generation (see table 2), 2^nd ^region (aa. 671-684 LAANDSERKNSWEP) contains N-glycosylation site

MORC3	Expressed in adult human brain [21]	Possible region cross (aa 665-696 DAVILPSCVEAEAKIHETQETTDKSADDAGC) similar to mouse Btnl2 and KIF21B

SIM2	Long isoform expressed in adult mouse brain particularly expressed in amygdala, hippocampus and thalamus, expression in embryonic and fetal brain, short isoform not expressed in adult brain [39-41]	Possible region (long form aa 613-624 GAAPAASGLAC) predicted low antigenicity

DSCR3/DCRA	Expressed in human and mouse adult brain [21,42]	No human specific region

DYRK1A	Expression in adult mouse and human brain [43-45]	No human specific region

KCNJ6/GIRK2	Expressed in subset of cells throughout mouse brain [46-48]	N/A

ETS2	Expressed in human and mouse brain [18,49]	No human specific region

PSMG1/DSCR2	Expressed in adult mouse brain [18,50]	No human specific region

B3GALT5	Expressed in adult human and mouse brain [18,51]	1 region used for antibody generation (see table 2)

PCP4/PEP-19	Expressed restricted to cerebellum and olfactory bulb in adult mouse and caudate-putamen in human [52-54]	No human specific region

DSCAM	Expressed in adult mouse and human brain [55,56]	No human specific region

BACE2	Expressed in adult mouse and human brain but at low levels [18,57-60]	Human specific region of 396 aa isoform (aa380-396 LQCLKFPGLSQQRM) predicted low antigenicity

UMODL1	Expression in embryonic mouse brain restricted to olfactory and vomeronasal neurons [61]	N/A

ABCG1	Expressed in adult human and mouse brain [21,62,63]	No human specific region

WDR4	Basal expression in adult tissues only high expression in fetal tissues [64]	N/A

PKNOX1/PREP1	Expressed in adult human and mouse brain [65,66]	No human specific region

CBS	Expressed in astrocytes and Bergmann glial cells only in adult mouse brain [67]	N/A

U2AF1	Expressed in adult mouse and human brain [21,68]	No human specific region

CSTB	Expressed in adult mouse and human brain (astrocytes and neurons) [18,21,69]	No human specific region

NNP1/RRPIB	Ubiquitously expressed in all human tissues [21,70]	2 regions used for antibody generation (see table 2)

AGPAT3	Expressed in adult mouse brain [71]	No human specific region

TRAPPC1/TMEM1	Expressed in adult human brain [21]	No human specific region

PWP2/PWP2H	Ubiquitously expressed in human adult tissue [72]	No human specific region

PFKL	Expressed in adult brain [21,73]	No human specific region

TRPM2	Expressed in human and mouse brain also in microglia cell lines and cultured neurons [74-77]	1 region used for antibody generation (see table 2)

PTTG1IP	Expressed in human brain [21,78]	1 possible region (aa 1-29 MAPGVARGPTPYWRLRLGGAALLLLLIPV) putative signal sequence [14]

ADARB1/RED1	Expressed in adult mouse and human brain [79-82]	1 region used for antibody generation (see table 2)

FTCD	Expressed in fetal human brain and in numerous mammalian cells types [83,84]	2 possible human specific regions (long isoform aa 423-446 GGPTGGSEAGSLCAADAGGDGGLA and aa 465-495 PPGGQSPGDGRVWRIFQRAHQPEGHHRRGI)

LSS	Expressed in adult mouse brain [18]	No human specific region

S100Beta	Expression in adult mouse and human brain (particularly astrocytes and spinal, medullar, pontine and deep cerebellar neurons) [18,21,85]	No human specific region

PRMT2	Expressed in human adult brain [21,86]	No human specific region

Evidence for expression in adult brain is listed in column two, including evidence for restriction of expression to a subset of cells. In column three candidate regions with moderate or low homology with their mouse ortholog are identified and any rational for not using the region to attempt to generate an antibody is described.

The secondary structure and accessibility of these low homology regions were modelled using PHD and PROF programmes that were accessed from the Predict Protein website http://cubic.bioc.columbia.edu/predictprotein/. Additionally, the regions were checked against published protein structures to confirm accessibility. The antigenicity of sequences was also estimated using the method of Jameson and Wolf which combines indicators of hydropathy, secondary structure and structural flexibility [12]. Candidate sequences were also checked for consensus sequences for posttranslational modifications including signal sequence cleavage, glycosylation, phosphorylation, and myristoylation using algorithms available from the Predict Protein website [13,14].

Candidate regions that were predicted to be accessible, not post-translationally modified, and exhibited a moderate/high antigenicity index, were checked for similarity with mouse proteins using blastp http://blast.ncbi.nlm.nih.gov/Blast.cgi. Those that were highly similar to mouse proteins were discarded as candidates. Ten candidate polypeptide sequences in eight candidate proteins were identified: an RNA editase (ADARB1), a Golgi-resident galactosyltransferase (B3GAL-T5) (two sequences), a potential neurodevelopmental protein (DOPEY2), the Golgi enzyme formimidoyltransferase-cyclodeaminase (FTCD), an RNA processing enzyme (RRP1) (two sequences), superoxide dismutase 1 (SOD1), a cation membrane channel (TRPM2) and a histone deubiquitinase (USP16).

**Figure 1:**
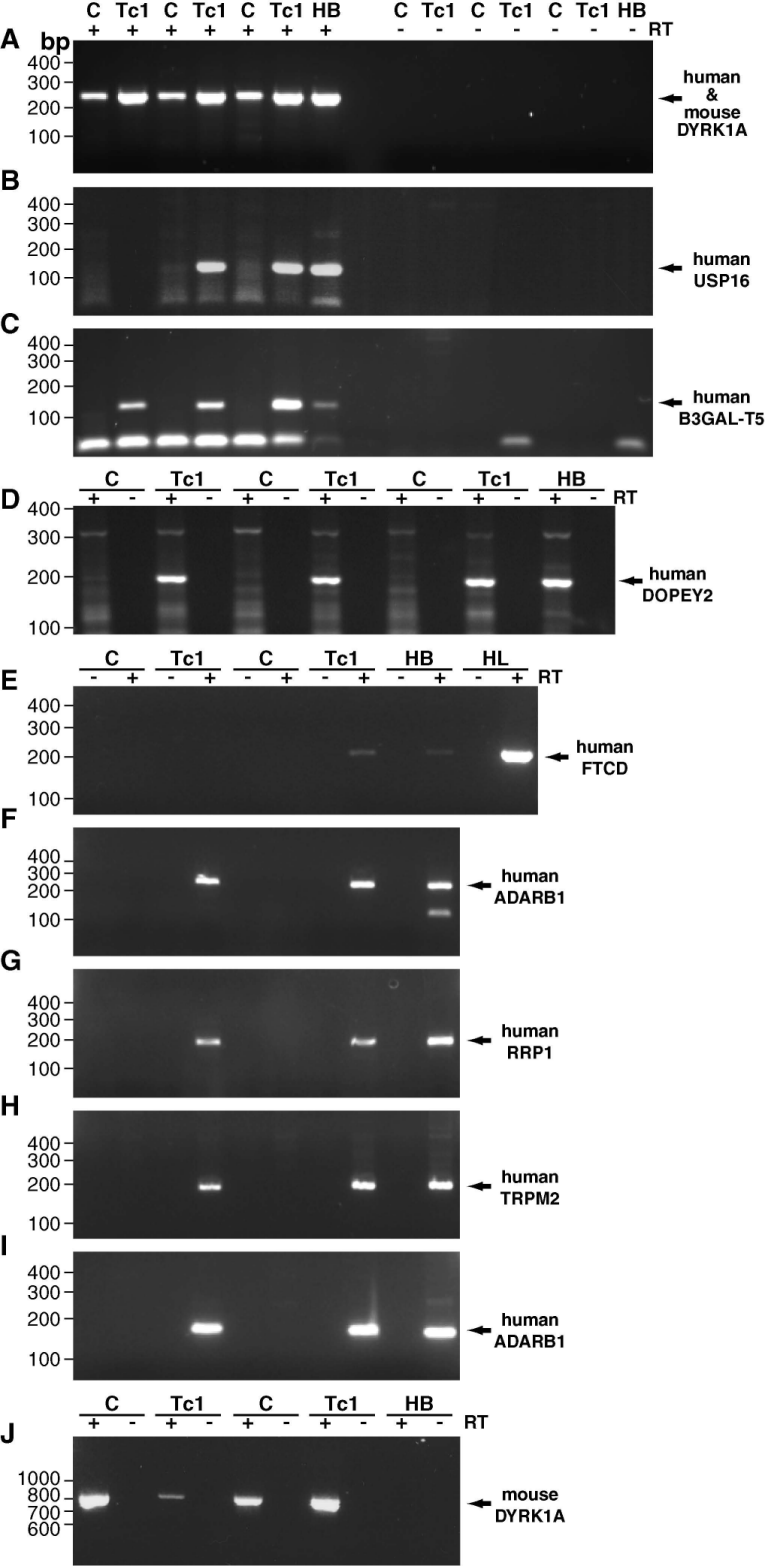
**Expression of candidate Hsa21 genes in the Tc1 adult brain**. To determine if the candidate genes were expressed, RT-PCR was undertaken using primers against (B) *USP16 *(129 base pair product), (C) *B3GAL-T5 *(138 base pair product), (D) *DOPEY2 *(192 base pair product), (E) *FTCD *(207 base pair product), (F) *ADARB1*-primer set 1 (134 or 254 base pair product), (G) *RRP1 *(173 base pair product) (H) *TRPM2 *(181 base pair product) and (I) *ADARB1*- primer set 2 (156 base pair product) was undertaken. Primers against (A) human and mouse *DYRK1A *(235 base pair product) and (J) mouse *Dyrk1a *(793 base pair product) were used as controls. RNA from Tc1 adult mouse brains (Tc1), non-transchromosomic littermate control adult brains (C), human adult brain (HB) and human adult liver (HL) was used as indicated.

Expression of *ADARB1*, *B3GAL-T5*, *DOPEY2*, *FTCD, RRP1*, *TRPM2 *and *USP16 *was investigated by RT-PCR. Total RNA was isolated from adult Tc1 mouse brain and non-transchromosomic littermate control brain, and subjected to RT-PCR (n = 5). Significant expression of *FTCD *could not be detected in human or Tc1 brain (Figure 1D). Therefore the two identified FTCD polypeptide sequences were discarded as potential candidates against which to raise an antibody. The expression of the other genes of interest was confirmed in the Tc1 brain (Figure 1). Elevated expression of SOD1 in the Tc1 brain had been previously demonstrated by western blot [2].

Production, conjugation of the selected peptides to Keyhole limpet haemocyanin (KHL) and injection of the KHL-peptides into New Zealand Rabbits was undertaken (21st Century Biochemicals). In the case of B3GAL-T5 and RRP1 a mixture of two peptides were injected into each rabbit (Table 2). Sera isolated from the rabbits after the fifth, sixth and seventh KHL-peptide boost was affinity purified against the peptide. Sera from the rabbits challenged with B3GAL-T5 and RRP1 peptides were affinity purified against both peptides separately.

**Table 2:** List of Hsa21 Genes and peptides used to immunise rabbits.

Candidate Protein	Molecular weight of protein (kDa)	Peptide used to immunise rabbits (protein accession numbers)	Mean antigenicity index (Jameson and Wolf)
Double-stranded RNA-specific editase 1 (ADARB1)	80	Acetyl-CNHGSLQPRPPGLLSDPS-amide (ENSP00000374512, amino acid 478-495)	1.088889

B-1,3-galactosyltransferase 5 (B3GAL-T5)	36	**A**. Acetyl-KERMVKGKQLKTF**C**-amide (ENSP00000343318, ENSP00000369994, ENSP00000369992, ENSP00000381699, ENSP00000403209 amino acid 82-93)	0.720833
		**B**.Acetyl-**C**AAETKEVDQESQRHGDI-amide (ENSP00000343318, ENSP00000369994, ENSP00000369992, ENSP00000381699, ENSP00000403209, amino acid 103-119)	1.547647

Protein Dopey 2 (DOPEY2)	258	Acetyl-CFRPVKQRYSVRNSVS-amide (ENSP00000382104, amino acid 455-470)	1.244375

Ribosomal RNA processing protein 1 homolog A (RRP1)	53	**A**. Acetyl-GDALSQKRSEKPPAGSI**C**-amide (ENSP00000291569, amino acid 257-275)	1.745
		**B**. Acetyl-**C**GARQRRRTPRPLTSARAKA-amide (ENSP00000291569, amino acid 428-446)	1.839474

Transient receptor potential cation channel subfamily M member 2 (TRPM2)	165, 177	Acetyl-**C**SWRLQ[Abu]PFGNNDKQESL-amide (ENSP00000381023, ENSP00000300481, ENSP00000300482, ENSP00000393982, ENSP00000381026, amino acid 43-59)	1.105882

Superoxide dismutase 1 (SOD1)	16	Acetyl-FEQKESNGPVKVWGSIC-amide (ENSP00000270142, amino acid 21-36, ENSP00000374645, amino acid 6-17)	0.9875

Ubiquitin carboxyl-terminal hydrolase 16 (USP16)	94	CSTEEVDMKNINMDNDLEV-amide (ENSP00000382857, ENSP00000382858, ENSP00000334808, amino acid 530-548)	0.930556

Added conjugating cysteine are highlighted in bold, amino acids that are only found in human sequence are underlined. Abu, abbreviation for amino-n-butyric acid, used to substitute for an internal cysteine residue.

### Antibodies that recognise a Tc1 Hsa21 specific protein

#### RRP1

**Figure 2:**
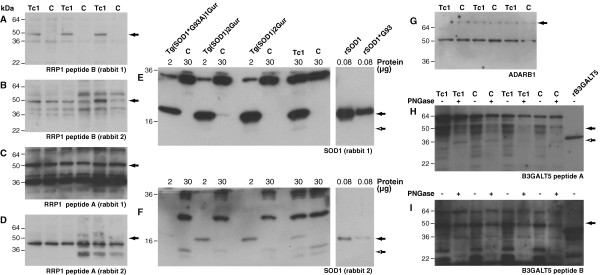
**Western blot of total brain proteins probed with affinity purified rabbit polyclonal antibodies**. Total brain proteins from Tc1 adult mice (Tc1+), non-transchromosomic littermate control mice (C), transgenic mice that express wild type human SOD1 (Tg(SOD1)2Gur), transgenic mice that express mutant human SOD1 (Tg(SOD1*G93A)1Gur)and non-transgenic littermate control mice (control), recombinant human SOD1 wild-type (rSOD1) and human SOD1 G93A (rSOD1*G93A) mutant protein and human recombinant B3GALT5 (amino acids 29-128) conjugated to GST were used as indicated. To compensate for the high expression level of the human SOD1 transgene in the Tg(SOD1)2Gur and Tg(SOD1*G93A)1Gur mice a lower amount of total brain proteins were loaded per lane as indicated. Western blots were probed with affinity purified anti-bodies raised against (A) RRP1 peptide B (9644-B), (B) RRP1 peptide B (9643-B), (C) RRP1 peptide A (9644-A), (D) RRP1 peptide A (9643-B), (E) SOD1 peptide (9638), (F) SOD1 peptide (9637), (G) ADARB1 peptide (9528), (H) B3Gal-T5 peptide A (9598-A) and (I) B3Gal-T5 peptide B (9598-B). Closed arrows indicate band of interest, (A-I), open arrows indicate potential mouse SOD1 band (E and F) and 36 kDa band in PNGase treated samples (H).

**Figure 3:**
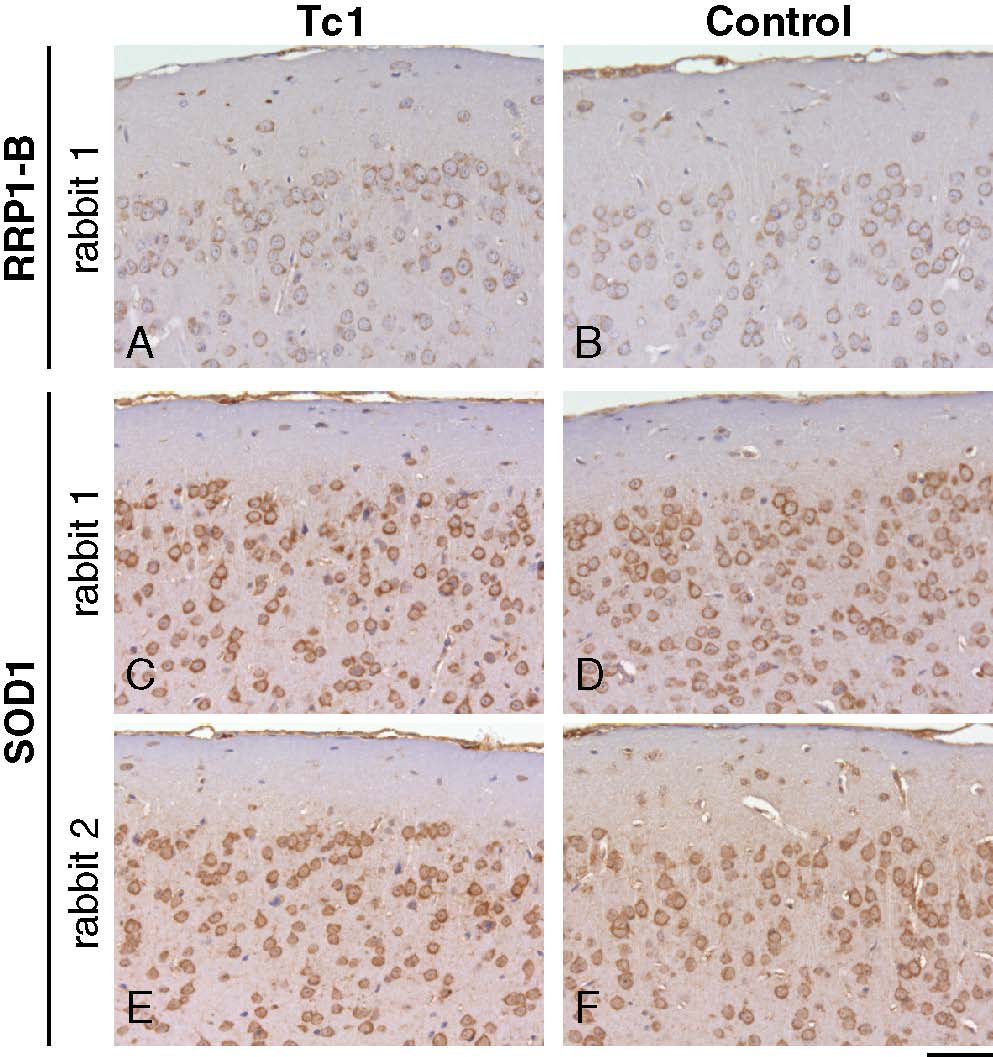
**Affinity purified anti-RRP1 and SOD1 antibodies do not specifically label cells in the Tc1 mouse model**. A similar pattern and intensity of staining is observed in adult Tc1 (A, C, E) and non-transchromosomic littermate control (B, D, F) cortical brain sections stained with affinity purified rabbit polyclonal antibodies generated against (A and B) RRP1 peptide B (9644-B), (C and D) SOD1 peptide (9638) and (E and F) SOD1 peptide (9637). Paraffin embedded sections were pretreated with protease prior to incubation with rabbit polyclonal antibodies, nuclei were counter-stained with haematoxylin. Scale bar = 50 μm.

One of the anti-RRP1 antibodies (9644-B), which was purified against peptide B, recognised a 50 kDa band on western blots of Tc1 total brain proteins; consistent with the predicted molecular weight of RRP1 (Figure 2A). A similar band was not observed in non-transchromosomic control mice, indicating that this antibody may specifically react with human RRP1. RRP1 peptide sequence B is unique to the human protein and is not found in mouse RRP1. In addition to the Tc1 specific band a number of weaker additional bands were observed in samples of Tc1 and non-Tc1 total brain proteins. These are likely to represent non-specific interaction of the polyclonal antibody with other brain proteins. Despite the relative specificity of the 9644-B antibody on western blot, a similar pattern and intensity of staining was observed on Tc1 and non-transchromosomic control mouse whole brain sections; intracellular staining was observed through-out the brain in both Tc1 and control non-transchromosomic mice (Figure 3A and 3B). Therefore, although 9644-B may be a suitable antibody for western blot studies of RRP1, it cannot be used to identify Hsa21 positive cells in the brains of Tc1 mice.

Affinity purified antibody raised against RRP1 peptide B purified from the second rabbit (9643-B) did not recognise a Tc1 specific band (Figure 2B). A 50 kDa protein was weakly detected using this antibody in samples of Tc1 and control mouse brain; however, peptide B does not share any homology with mouse RRP1 therefore the 50 kDa band detected after probing with this antibody is highly unlikely to be RRP1.

An antibody affinity purified against RRP1 peptide A (9644-A) did recognise a band consistent with the molecular weight of RRP1 in samples of both Tc1 and control brain (Figure 2C). Five of the nineteen amino acids of peptide A are homologous with the mouse RRP1 protein sequence including a sequence (K*PA) with high predicted antigenicity. Therefore the antibody purified against peptide A may recognise both mouse and human RRP1 and therefore is not useful to identify Hsa21 positive cells in the Tc1 model. An antibody affinity purified against peptide A from the other rabbit (9643-B) did not consistently recognise a band corresponding to the molecular weight of RRP1 (Figure 2D). This suggests that RRP1 peptide A is not a reliable antigen for the production of rabbit polyclonal antibodies.

### Antibodies that did not recognise a Tc1 unique product

#### SOD1

Immunisation with a single SOD1 peptide generated anti-SOD1 antibodies (9638 and 9637) that recognised a Tc1 specific band on western blots of total brain protein (Figure 2E and 2F). The size of the bands recognised is consistent with the known molecular weight of the SOD1 monomer (16 kDa). These antibodies also detected a band of a comparable molecular weight in samples of total brain proteins isolated from transgenic mice that over-express wild-type or mutant (SOD1^G93A^) human SOD1 and in samples of recombinant human SOD1 (wild-type or SOD1^G93A^) (gift of Ruth Chia) (Figure 2E and 2F). The 16 kDa band was not observed in samples of brain from non-transchromosomic control mice. However, after long exposures a weak band that was smaller than the predominant 16 kDa band was detected by both 9637 and 9638 in Tc1 and control mouse brain samples. This smaller band may be mouse SOD1; thus antibody 9637 and 9638 may weakly cross-react with mouse SOD1. Moreover, these antibodies generated an intracellular staining pattern of similar intensity on Tc1 and non-transchromosomic control mice brain sections, which were either paraffin-embedded or cryopreserved (Figure 3C-F, data not shown). The antibody does not recognise cells specifically in the Tc1 brain and therefore cannot be used to identify these Hsa21 positive cells in our mouse model for future studies. This result may occur because the polyclonal antibodies generated recognise non-SOD1 proteins and weakly cross-react with mouse SOD1 in both Tc1 and control brain, or that the antibodies generated only recognise denatured human SOD1. We have previously tested whether a number of commercially available anti-SOD1 antibodies specifically label cells in Tc1 brain sections and found that these antibodies were not specific (data not shown).

#### ADARB1

An affinity purified antibody (9528) that reacted weakly with a band consistent with the known molecular weight of the protein, 80 kDa, was isolated from one rabbit injected with the ADARB1 peptide (Figure 2G). However, this band was observed in samples of total brain proteins from both Tc1 and non-transchromosomic control mice. As ADARB1 peptide sequence used to challenge the rabbits was unique to human ADARB1 and not found in mouse, the protein recognised by this antibody is unlikely to be ADARB1. No signal consistent with the molecular weight of ADARB1 was observed when western blots of total brain proteins were probed with affinity purified antibody generated from the second rabbit (9529), which was challenged with ADARB1 peptide (data not shown).

#### B3GALT5

Affinity purified antibodies raised against B3GAL-T5 peptides were used to probe western blots of total brain proteins from Tc1 and control mice and recombinant glutathione-S-transferase (GST) tagged human B3GAL-T5 (amino acids 29-128, Abnova). Recombinant human B3GAL-T5 was detected using both antibodies (Figure 2H and 2I). A predominant band of 64 kDa and weaker bands of around 50 kDa were detected in western blots of Tc1 and control samples probed with antibodies affinity purified against peptide A (9598-A) (Figure 2H). A predominant band of 50 kDa and weaker bands of 64, 36 and approximately 28 kDa were detected in western blots of samples of total brain proteins from Tc1 and control mice that were probed with antibodies affinity purified against peptide B (9598-B) (Figure 2I). The molecular weight of human B3GAL-T5 is 36 kDa. However, B3GAL-T5 contains three N-glycosylation sequences (amino acids 130, 174 and 231) that may be occupied in vivo. Indeed in COS-7 cells a variety of B3GAL-T5 glycoforms of between 37-50 kDa are detected by western blot [15]. To investigate if the protein bands detected in samples of Tc1 and control brain are glycosylated forms of B3GAL-T5 samples of Tc1 and control brain proteins were treated with PNGase F, an enzyme that cleaves protein-attached N-linked glycans, before western blotting. De-glycosylation of endogenous proteins was confirmed by checking that the glycoprotein PrP exhibited the expected size shift after PNGase F treatment (data not shown). Enrichment of a 36 kDa protein was observed in Tc1 and control brain samples after treatment PNGase F on western blots probed with the antibody affinity purified against peptide A (9598-A), consistent with this antibody recognising endogenous B3GAL-T5 (Figure 2H). No enrichment in a 36 kDa band was observed in the brain samples treated with PNGase F that were probed with the antibody affinity purified against peptide B (9598-B) (Figure 2I). This result suggests that the 50 kDa protein recognised by antibody 9598-B is not a glycosylated form of B3GAL-T5.

#### DOPEY2, TRPM2 and USP16

Affinity purified rabbit polyclonal antibodies raised against DOPEY2 and TRPM2 and USP16 peptides did not react with a band of the predicted molecular weight, in western blots of Tc1 and non-transchromosomic control total brain proteins (data not shown). In addition the pattern and intensity of staining observed in Tc1 and non-transchromosomic control paraffin-embedded or cryopreserved brain sections was similar, indicating that that these antibodies do not recognise a Hsa21 specific product (data not shown).

## Discussion

In order to specifically detect cells carrying Hsa21 in our Tc1 mice, we carried out extensive literature searches of both commercial and basic research resources and were unable to find suitable antibodies that could be used on fixed tissues and primary cell cultures. Many antibodies to Hsa21 derived proteins exist, but none that we could find specifically recognised Hsa21 positive cells in Tc1 mouse brain sections and not control non-transchromosomic mouse sections. Therefore we attempted to generate Hsa21 antibodies that we could use to identify Hsa21 carrying cells in our model.

From bioinformatics analysis, we identified eight genes which were present in the Tc1 mouse and which might make suitable candidates for further analysis. One of these, *FTCD*, was not expressed in brain and so we generated eighteen different antibodies raised against amino-acid sequences identified from the remaining seven genes, selecting only sequences which were divergent between mouse and human, and likely to be moderately/highly antigenic.

We generated a panel of antibodies, of which one antibody (9644-B) raised against RRP1 appeared to be human specific on western blots, although proved unsuitable for immunohistochemistry and two new antibodies raised against SOD1 (9638 and 9637) that appear to preferentially recognise human SOD1 on western blots, but do not recognise Hsa21 positive cells in Tc1 brains by immunohistochemistry.

## Conclusion

Having surveyed 295 genes on Hsa21 we are left with three antibodies that we can use for western blot analysis that will preferentially bind to human protein, and none that will work by immunohistochemistry. This illustrates the difficulty of making antibodies that only recognise a specific human protein but not its mouse homologue, even with extensive knowledge of the genes available, their likely antigenicity and the degree of conservation between mouse and human. We will now go on to other methods for detecting Hsa21 in tissue sections and cultured cells, and we note that the antibodies we have generated are available to interested laboratories.

## Methods

### Animal Welfare

Mice were housed in controlled conditions in accordance with guidance issued by the Medical Research Council in *Responsibility in the Use of Animals for Medical Research *(1993) and all experiments were carried out under License from the UK Home Office.

### DNA extraction and Genotyping

DNA was extracted from tail tip (approximately 3mm) from all samples analysed. Tail tip is lysed overnight using Proteinase K digestion in nuclei lysis buffer (Promega), plus 0.12 M EDTA at 55°C. Proteins are precipitated from the resultant lysate by addition of protein precipitation solution (Promega), DNA is then precipitated with isopropanol and resuspended in DNase free water. Tc1 mice were genotyped using PCR (Tc1 specific primers f: 5'-GGTTTGAGGGAACACAAAGCTTAACTCCCA-3' r: 5'-ACAGAGCTACAGCCTCTGACACTATGAACT-3', control primers f: 5'-TTACGTCCATCGTGGACAGCAT-3' r: 5'-TGGGCTGGGTGTTAGTCTTAT-3'). Tc1 mice were taken from a colony maintained by mating Tc1 females to F1(129S8 × C57BL/6) males. Both SOD1 transgenics were taken from colonies maintained by crossing male transgenics to female C57BL6/J (Jackson Laboratories, Bar Harbour). SOD1 transgenic mice (Tg(SOD1)2Gur, Jackson and Tg(SOD1*G93A)1Gur; Jackson Laboratories, Bar Harbour) were genotyped by PCR (SOD1 specific primers f: 5'-CATCAGCCC TAATCCATCTGA-3' r: 5'-CGCGACTAACAATCAAAGTGA-3', control primers f: 5'-CTAGGCCACAGAATTGAAAGATCT-3' r: 5'-GTAGGTGGAAATTCTAGCATCATC-3').

### RNA extraction and RT-PCR

RNA was extracted from whole brains from 6-10 week old Tc1 and age and sex matched non-transchromosomic controls. Total RNA was extracted using TRIzol reagent (Invitrogen), precipitated as per manufactures instructions and resuspended in DNase-free water. Amounts of RNA were equalised and cDNA was generated using a standard reverse-transcription protocol using random primers (Promega), Superscript II (Invitrogen), First Strand Buffer (Invitrogen) and dNTPs (Promega). PCR using primers which amplify a PCR product from both mouse Dyrk1A and human DYKR1A (f: 5'-GGAGAGACTTCAGCATGCAAAC-3' r: 5'-GCTGGGTCACGGAAGGTTTG-3') or mouse DYRK1A (f: 5'-CAAGAAAACAGCTGATGAAGG-3' r: 5'-AGCCCCTTGTCTCATCGC-3') were used to check cDNA. PCR using primers designed to raised a product against human but not mouse *FTCD *(f: 5'-GAATGCGTCCCCAACTTTTCG-3' r: 5'-GTCGATAAGTCGGGAAGCTAC-3'), *USP16 *(f: 5'-AAGCCTTCAGTTTGGCTG-3' r: 5'-GTCCAAACTAAGAACCAGAC-3'), *DOPEY2 *(f: 5'-ACCTGAGGTACTCCTTGTTG-3' r: 5'-CCAGGAGAGGAAATAACCCG-3'), *TRPM2 *(f: 5'-GTTCGTGGATTCCTGAAAAC-3' r: 5'-TCCAAGTGCTGCTCATGC-3' and f: 5'-TGGCCGTCAGCGTCCACTTC-3' r: 5'-TAGTGAGCCCCGAACTCAGC-3'), *B3GAL-T5 *(f: 5'-CACTGTGGCTTTAGCTTTCAAAC-3' r: 5'-GGATTTAGACTGTACATGC-3'), *ADARB1 *(f: 5'-TTTAGGCTGAAGGAGAATGTC-3' r: 5'-CCTCTTGCTTTACGATTTGGG-3' and f: 5'-GTCTCGCTCTTACACCCAG-3' r: 5'-CCTCTTGCTTTACGATTTGGG-3') and *RRP1 *(f: 5'-TCCCTGAAGATGAGATCCCAG-3' r: 5'-TACACCCCTCCTCCTGCTC-3') were used to check the expression of these genes from Hsa21.

### Western blotting

Whole brain from Tc1, Tg(SOD1)2Gur, Tg(SOD1*G93A)1Gur and aged and sex matched control non-transgenic mice was homogenized in 9 volumes of RIPA Buffer (150 mM sodium chloride, 50 mM Tris, 1% NP-40, 0.5% sodium deoxycholate, 0.1% sodium dodecyl sulfate) or phosphate buffered saline plus complete protease inhibitors (PBS) (Roche Applied Science) by mechanical disruption using a dounce homogenizer. Total protein content was determined using the DC protein Assay (Biorad). Samples that were homogenized in PBS were treated with PNGase F (15 U/μg protein) (New England Biolabs) for 3 hours shaking at 37°C to cleave N-linked glycans. The resultant total brain protein and recombinant protein samples were denatured in SDS denaturing buffer (Invitrogen) and β-mercaptoethanol for 10 minutes at 100°C, prior to separation by SDS-PAGE gel electrophoresis using precast 16% or 4-20% Tris-glycine gels (Invitrogen). Proteins were transferred to PVDF membrane prior to blocking in 5% milk PBS for 1 hour before incubating over-night with primary antibody at 4°C. Membranes were then incubated with an anti-rabbit secondary antibody (Sigma-Aldrich) conjugated to alkaline phosphatase prior to development with CDP-Star (Roche Applied Sciences) and exposure to X-ray film. See-Blue plus 2 (Invitrogen) was used as a molecular weight marker.

### Immunohistochemistry

Whole Tc1 and non-transchromosomic control mouse brain was fixed by immersion in 10% buffered formal saline (Pioneer Research Chemicals). Following further washing for 24 hr in 10% buffered formal saline, tissue samples were processed and embedded in paraffin wax. Sections were cut at a thickness of 4 μm. Alternatively brains were protected in Tissue-Tek (Siemens Healthcare Diagnostics) and frozen by immersion in isopentane chilled with liquid nitrogen. Frozen sections were cut at a thickness of 10 μm on a cryostat and air dried prior to staining. Paraffin-embedded sections were pretreated by protease digestion. Staining with the rabbit polyclonal antibodies was undertaken using a Ventana automated immunohistochemical staining machine (Ventana Medical Systems, Tuscon, AZ, USA) as described previously [16]. A biotinylated-anti-rabbit IgG secondary antibody (*i*View SA-HRP, Ventana Medical Systems) was used before development with 3'3 diaminobenzidine tetrachloride as the chromogen (iView DAB, Ventana Medical Systems). Haematoxylin was used as the counter-stain.

## Competing interests

The authors declare that they have no competing interests.

## Authors' contributions

FW carried out the bioinformatic searches, immunohistochemistry and some RT-PCRs and western blots, analysed the data and assisted in drafting the manuscript. OS carried out the some RT-PCRs and western blots and assisted in drafting the manuscript. JL and SB assisted with IHC data collection and analysis. EF and VT conceived the study, and participated in its design and coordination and helped to draft the manuscript. All authors read and approved the final manuscript
